# Focal persistence and phylodynamics of Heartland virus in Georgia

**DOI:** 10.1093/ve/veaf064

**Published:** 2025-08-23

**Authors:** Stephanie Bellman, Nima Shariatzadeh, Tyshawn Ferrell, Audrey Long, Leah Aeschleman, Ellie Fausett, Tim Walsh, Seana Cleary, Isabella Roeske, Erick Ojeda, Madison Schwab, Hannah Dakanay, Sam R Telford III, Heidi K Goethert, Gonzalo Vazquez-Prokopec, Anne Piantadosi

**Affiliations:** Gangarosa Department of Environmental Health, Rollins School of Public Health, Emory University, 1518 Clifton Road Northeast, Atlanta, GA 30322, United States; Department of Environmental Sciences, Emory University, 201 Dowman Drive, Atlanta, GA 30322, United States; Department of Pathology and Laboratory Medicine, Emory University, School of Medicine, 100 Woodruff Circle, Atlanta, GA 30322, United States; Department of Pathology and Laboratory Medicine, Emory University, School of Medicine, 100 Woodruff Circle, Atlanta, GA 30322, United States; Department of Pathology and Laboratory Medicine, Emory University, School of Medicine, 100 Woodruff Circle, Atlanta, GA 30322, United States; Department of Microbiology and Immunology, Emory University, 201 Dowman Drive, Atlanta, GA 30322, United States; Graduate Program in Biochemistry, Cell, and Developmental Biology, Emory University, 201 Dowman Drive, Atlanta, GA 30322, United States; Department of Environmental Sciences, Emory University, 201 Dowman Drive, Atlanta, GA 30322, United States; Department of Environmental Sciences, Emory University, 201 Dowman Drive, Atlanta, GA 30322, United States; Gangarosa Department of Environmental Health, Rollins School of Public Health, Emory University, 1518 Clifton Road Northeast, Atlanta, GA 30322, United States; Department of Environmental Sciences, Emory University, 201 Dowman Drive, Atlanta, GA 30322, United States; Gangarosa Department of Environmental Health, Rollins School of Public Health, Emory University, 1518 Clifton Road Northeast, Atlanta, GA 30322, United States; Department of Environmental Sciences, Emory University, 201 Dowman Drive, Atlanta, GA 30322, United States; Gangarosa Department of Environmental Health, Rollins School of Public Health, Emory University, 1518 Clifton Road Northeast, Atlanta, GA 30322, United States; Department of Environmental Sciences, Emory University, 201 Dowman Drive, Atlanta, GA 30322, United States; Department of Environmental Sciences, Emory University, 201 Dowman Drive, Atlanta, GA 30322, United States; Gangarosa Department of Environmental Health, Rollins School of Public Health, Emory University, 1518 Clifton Road Northeast, Atlanta, GA 30322, United States; Department of Pathology and Laboratory Medicine, Emory University, School of Medicine, 100 Woodruff Circle, Atlanta, GA 30322, United States; Graduate Program in Population Biology, Ecology, and Evolution, Emory University, 201 Dowman Drive, Atlanta, GA 30322, United States; Department of Pathology and Laboratory Medicine, Emory University, School of Medicine, 100 Woodruff Circle, Atlanta, GA 30322, United States; Department of Infectious Disease and Global Health, Cumming School of Veterinary Medicine, Tufts University, 200 Westboro Road, Grafton, MA 01536, United States; Department of Infectious Disease and Global Health, Cumming School of Veterinary Medicine, Tufts University, 200 Westboro Road, Grafton, MA 01536, United States; Department of Environmental Sciences, Emory University, 201 Dowman Drive, Atlanta, GA 30322, United States; Department of Pathology and Laboratory Medicine, Emory University, School of Medicine, 100 Woodruff Circle, Atlanta, GA 30322, United States; Department of Medicine, Division of Infectious Diseases, Emory University, School of Medicine, Atlanta, GA 30322, United States

**Keywords:** Heartland virus, phylodynamics, *Amblyomma americanum*, Georgia, full genome sequencing

## Abstract

Heartland virus (HRTV) is an emerging tick-borne virus associated with severe illness in the USA. There are large gaps in knowledge of HRTV diversity, evolution, and transmission due to a paucity of HRTV-positive samples and genome sequences. We identified a focal site of HRTV-positive *Amblyomma americanum* ticks in central Georgia and developed a novel multiplex-amplicon sequencing assay to generate full HRTV genome sequences. By screening over 21 000 field-collected ticks from 2021 to 2023, we identified six positive pools. Five were collected from the site in central Georgia where our group first detected HRTV-positive ticks in 2019 and one from a site in western Georgia ~175 km away. The HRTV genome sequences from Georgia were highly related, even across this distance and over five years. Reference HRTV genome sequences from across the USA were also geographically clustered. Time-scaled phylogenetic analysis suggested a recent spread of HRTV in the USA, with all available sequences sharing a common ancestor within the last 75 years, since the mid-1900s, and sequences from Georgia sharing a common ancestor within the last 15 years, since 2010. Our observed spatial clustering of HRTV and the high degree of genetic conservation in our persistent focus suggest the importance of small spatial dynamics in HRTV transmission ecology.

## Introduction

In 2009, two farmers in northwestern Missouri presented with symptoms similar to ehrlichiosis and were found to be infected with a novel pathogen, Heartland virus (HRTV) (McMullan *et al.* 2012). Originally categorized as a *Phlebovirus*, HRTV was recategorized to the genus *Bandavirus* in the family *Phenuiviridae,* order *Bunyavirales* in 2020 (Abudurexiti *et al.* 2019). HRTV is vector-borne and transmitted by the lone star tick (*Amblyomma americanum*), whose range spans the majority of the eastern half of the USA (Brault *et al.* 2018; Centers for Disease Control 2025). To date, HRTV has been associated with over 60 cases of human disease in 14 US states and has an observed case fatality rate of 5%–10% (Dembek *et al.* 2024).

HRTV, like other bunyaviruses, has a segmented negative-sense single-stranded RNA genome with three segments (McMullan *et al*. 2012). The L segment encodes the RNA-dependent RNA polymerase (RdRp) and is 6.4 kb long. The M segment encodes two viral glycoproteins (Gn, Gc) and is 3.4 kb long. Lastly, the S segment employs an ambisense strategy to encode the nucleoprotein (Np) and nonstructural protein (NSs) in 1.7 kb. HRTV is most closely related to Dabie bandavirus (DBV) (previously known as Severe Fever with Thrombocytopenia Syndrome virus, SFTSV), which circulates in China, Japan, South Korea, Vietnam, and Taiwan (Casel *et al.* 2021). While different genotypes of DBV have been documented across Asia (Li *et al.* 2021) and have been associated with different pathogenicity in humans (Yun *et al.* 2020b), very little is known about the genotypic and phenotypic diversity of HRTV. This knowledge gap stems from limited surveillance, low HRTV prevalence in ticks (Savage *et al.* 2016; Savage *et al.* 2018; Dupuis 2nd *et al.* 2021), and few detected human cases (Centers for Disease Control 2024).

HRTV has been identified in *A. americanum* populations in eight states across the USA, spanning the Northeast, Southeast, and Midwest (Dupuis II *et al.* 2023). Prior surveillance studies for HRTV in ticks have been episodic, typically responding to newly discovered human cases, and continuing for one to two years (Savage *et al*. 2016; Savage *et al*. 2018; Dupuis 2nd *et al.* 2021; Aziati *et al.* 2023). Few studies have continued surveillance beyond 2 years, and there is very little HRTV genome sequence data available, creating a gap in understanding the enzootic transmission and evolutionary dynamics of HRTV.

The objective of this study was to begin to fill this knowledge gap through long-term surveillance at established HRTV-positive tick sites in central Georgia (GA), where our group initially detected HRTV in 2019 (Romer *et al.* 2022). In our prior study, we found a minimum infection rate of 0.46/1000 HRTV-positive *A. americanum* ticks at two locations where HRTV seropositivity was previously reported in deer populations (Clarke *et al.* 2018b) and adjacent to the county where a human case was reported (Centers for Disease Control 2024). In the present study, we build upon this by performing extended HRTV surveillance in GA across multiple years. We use a novel multiplex amplicon sequencing approach to generate full HRTV genome sequences and analyse phylogenetic and phylogeographic relationships of HRTV in GA and across the USA, gaining insight into potential mechanisms of virus transmission and evolution.

## Materials and methods

### Field collections

Ticks were collected from March to August 2021–23, corresponding to the seasonality of *A. americanum* in Georgia (Semtner and Hair 1973). Collection locations varied by year: in 2021, repeated sampling was conducted at the two sites where our group detected HRTV in 2019 (Romer *et al*. 2022); in 2022, sampling was performed in 43 locations across the state (Bellman *et al.* 2024); and in 2023, five locations with the highest density of *A. americanum* from the previous years were repeatedly sampled to maximize potential HRTV detection. Free flagging was conducted at each site according to previously described protocols (Fausett *et al.* 2024), with the addition of transect sampling during 2022 (Bellman *et al*. 2024). Ticks were transported live in plastic vials to the laboratory and microscopically identified with taxonomic keys (Keirans and Litwak 1989; Keirans and Durden 1998).

### Tick processing, RNA extraction, and qRT-PCR

Surface disinfection was conducted on ticks in field vials using a wash of 10% bleach for 5 min, 3% hydrogen peroxide for 5 min, and three rinses of distilled water for 5 min each. The ticks were pooled in groups of approximately five adults or 25 nymphs per species per site. Differing amounts of BA-1 diluent (1× medium 199 with Hanks balanced salt solution, 0.05 mole/L Tris buffer [pH 7.6], 1% bovine serum albumin, 0.35 g sodium bicarbonate/L, 100 μg/L streptomycin, 1 μg/ml amphotericin B) were added to each pool based on the number of ticks in the pool (1 ml for >1 adult or  >10 nymphs, 0.5 ml for 1 adult or  <10 nymphs) to preserve concentrations of the ground ticks in media. Pools were ground thoroughly using a 7 ml glass TenBroeck grinder (Fisher Scientific) with alundum bedding material (Fisher Scientific) as an abrasive.

Tick homogenates underwent RNA extraction (QIAmp TNA Extraction Kit) following the manufacturer’s protocol (Supplemental Methods). Extracted RNA was tested using quantitative reverse-transcriptase PCR (qRT-PCR) with primer-probe set 1 for the S segment of HRTV described (Savage *et al*. 2016) and the AgPath-ID One-Step RT-PCR kit (Thermo Fisher Scientific), per the manufacturer’s instructions (Supplemental Methods). Samples were tested in duplicate with positive controls [extracted HRTV virus stock (Romer *et al*. 2022)] and negative controls (water) on each plate. As a quality control check for RNA extraction, tick actin RT-qPCR was run in parallel using the Applied Biosystems Power SYBR Green RNA-to-C_T_ 1-step PCR Kit (Fisher Scientific) with primers, probes, and reaction conditions listed in the Supplemental Methods.

### Infection rate estimation

The minimum infection rate (MIR) of HRTV was estimated by site and year by dividing the number of positive pools at a location by the number of *A. americanum* of the same life stage collected at the site and year and multiplying by 1000.

### Heartland virus multiplex amplicon sequencing primer design

Multiplex amplicon sequencing primers for HRTV were designed with primalscheme (v1.4.1) (Quick *et al.* 2017) using an alignment of four (L) or five (M and S) complete reference HRTV sequences (Sayers *et al.* 2019). Primers that had high annealing temperatures (65°C or greater) or had SNPs expected to affect primer binding, as determined by comparison to additional partial genome sequences, were redesigned in Geneious Prime (v2022.2). A set of 36 L, 18 M, and 10 S primer pairs was created to cover the entire genome, with each primer pair amplifying an ~250 bp region. The HRTV-specific primer sequences were concatenated with a universal Nextera adapter sequence to allow Nextera XT indexing and amplification (Glenn *et al.* 2019; Matteson *et al.* 2023). Primers were synthesized by IDT, pooled, and tested using a previously sequenced HRTV-positive sample (Romer *et al*. 2022). Primer concentrations were optimized to ensure approximately equal coverage across the entire genome. Primer sequences and concentrations are listed in Supplemental Table S1, and primer bed files are listed in Supplemental Table S2.

### Heartland virus full-genome multiplex amplicon sequencing

RNA samples that tested positive for HRTV *via* RT-qPCR underwent heat-labile dsDNase treatment (ArticZymes), single-stranded cDNA synthesis (Fisher/Invitrogen), and multiplex PCR using Q5 Hot Start High-Fidelity Polymerase (New England Biolabs) using the custom-designed primers. The resulting amplicons were indexed using Nextera XT (Illumina) and quantified using the KAPA Universal Complete Kit (Roche). Libraries were pooled to equimolar concentration and sequenced with 250 bp paired-end reads on an Illumina MiSeq. Reads were filtered for quality, and adaptor sequences were removed, before undergoing reference-based assembly, all using viralrecon v. 2.6.0 (Patel *et al*. 2023) with reference sequences L: NC_024495, M: NC_024494, and S: NC_024496. Samples were processed alongside negative controls (water) and positive controls [HRTV-positive tick pools from our prior study (Romer *et al*. 2022)]. A more complete protocol for the laboratory and analysis methods can be found in the Supplemental Methods, and the sequencing metrics for each sample are listed in Supplemental Table S3.

### Phylogenetic analysis

Twelve reference sequences of HRTV L, M, and S segments present in GenBank as of 1 March 2024 were downloaded for analysis. Selection criteria included: related to a field-collected case (tick or human), no identical isolates (if an isolate was sequenced more than once, the earliest was selected), and >95% genome coverage. Newly generated sequences and reference sequences were aligned by segment in Geneious Prime (v2022.2.2) using MAFFT (v7.450) with default parameters (Katoh *et al.* 2002; Katoh and Standley 2013). Maximum-likelihood phylogenetic trees were generated for each segment using IQ-TREE (v2.3.5)(Minh *et al.* 2020), and model selection was performed using ModelFinder (Kalyaanamoorthy *et al.* 2017). The best fit substitution models according to the Bayesian Information Criterion (BIC) were: TIM3 + F + I for the L segment (proportion of invariable sites: 0.801), TPM2u + F + G4 for the M segment (gamma shape alpha: 0.269), and TPM3 + I for the S segment (proportion of invariable sites: 0.769). Trees were visualized, rooted on the RefSeq reference sequence for each segment (L: NC_024495, M: NC_024494, and S: NC_024496), and annotated using iTOL (Letunic and Bork 2021). Reassortment was assessed through three methods: (i) by visually comparing segment relationships across different clades within the trees, (ii) by testing aligned concatenated coding regions with the recombination detection program, RDP4 (Martin *et al.* 2015), and (iii) by comparing the trees using the R package dendextend (v1.18.1) (Galili 2015), with the step2side untangle function used to rotate branches and minimize entanglements.

### Assessment of temporal signal and Bayesian time-scaled phylogenetic analysis

To construct time-scaled phylogenies, the temporal signal of each segment’s coding region was first evaluated using TempEST (Rambaut *et al.* 2016), and one outlier (OK480062.1) was removed due to unbalanced residuals in the L segment (Supplemental Fig. S1); the corresponding S and M segments from the same sample were also removed (OQ688995.1 and OQ688994.1, respectively). Subsequently, the root-to-tip correlation function in TempEST provided evidence for a moderate temporal signal in all segments, with correlation coefficients of 0.63 for the L segment (*R*^2^ = 0.41, slope = 1.26E-3), 0.59 for the M segment (*R*^2^ = 0.35, slope = 2.82E-3), and 0.63 for the S segment (*R*^2^ = 0.40, slope = 1.62E-3). Temporal signal was formally evaluated using Bayesian evaluation of temporal signal (BETS) (Duchene *et al.* 2020) in BEAST2 (v2.7.6) (Bouckaert *et al.* 2014), using path sampling to compare heterochronous versus isochronous data, as well as comparing a strict clock to an optimized relaxed clock, and comparing a lognormal distribution and a gamma distribution for the constant population size prior, as has been done in prior studies (Bondaryuk *et al.* 2023; Vogels *et al*. 2023; Holtz *et al.* 2024). We found evidence for a temporal signal in the S segment, for which the best-fit model included heterochronous data, an optimized relaxed clock, and a constant population size with a gamma distribution prior (Supplemental Table S4). Using BETS, we did not find evidence for a temporal signal in the M or L segments.

For the S segment, model testing was performed by path sampling in BEAST2 (v2.7.6) to compare a coalescent constant population model versus Bayesian skyline versus Extended Bayesian Skyline, all with an optimized relaxed clock; the coalescent constant population was the best-fit model (Supplemental Table S5). For all model testing, including BETS, all steps were summarized using Path Sample Analyser (Baele *et al.* 2013; Baele and Lemey 2013; Bouckaert *et al.* 2019), and the log Bayes factor was calculated as the log difference in marginal likelihoods between each model and the best-fit model.

A final time-scaled phylogeny was constructed for the S segment using BEAST2 (v2.7.6) with an optimized relaxed clock with a lognormal distribution rate prior, a constant coalescent population with a gamma distribution population size prior, and an MCMC chain length of 500 million (Bouckaert *et al*. 2014). We used the best-fit nucleotide substitution model that was identified by IQ-TREE, TPM3. The log files were examined with Tracer v1.7.2 and confirmed to have ESS values > 200 for all parameters (Rambaut *et al.* 2018). Maximum clade credibility trees were generated with 10% burn-in using TreeAnnotator v2.7.6 (Bouckaert *et al*. 2014). All xml, log, and tree files are available at: https://github.com/Piantadosi-Lab/Focal-Persistence-and-Phylodynamics-of-Heartland-Virus-in-Georgia

### Geographic clustering analysis

The Mantel test (Diniz-Filho *et al.* 2013) was used to assess the relationship between geographic distance and genetic divergence. Because exact coordinates were not known for the reference sequences, the location was estimated using the finest resolution information present. For samples with only a state recorded for the source location in GenBank, the centre point of the most central county in the state was assigned, using USGS (USGS 2024) and Latitude.to (v1.64-im). For samples with a county and a state recorded for the source location in GenBank, the centre of the county listed for that sequence was assigned. For sequences associated with publications, the smallest resolution estimate was made based on recorded location information (Savage *et al.* 2013; Savage *et al*. 2018; Dupuis 2nd *et al.* 2021). Lastly, for the two initial Missouri human case sequences, Andrews county and Nodaway county centres were used (Savage *et al*. 2013). The Mantel test was run using the vegan package (v2.5-7) (Oksanen *et al.* 2020) in R (v4.0.4) using 1000 permutations.

### Host bloodmeal analysis

Nucleic acid extracted from HRTV-positive *A. americanum* pools and matched HRTV-negative *A. americanum* pools—containing a similar number of ticks, and the same life stage, collection site, and collection date—were tested by multiplex qPCR targeting retrotransposons of potential hosts, according to established protocols (Goethert 2021; Goethert *et al.* 2021). Species included in the assay were: mice, voles, rabbits, birds, shrews, squirrels/chipmunks, deer, skunks, and opossums. Any amplification seen by qPCR was treated as a positive for that host.

## Results

### Six Heartland virus–positive tick pools were detected across central and western Georgia, USA, from 2021 to 2023

**Figure 1:**
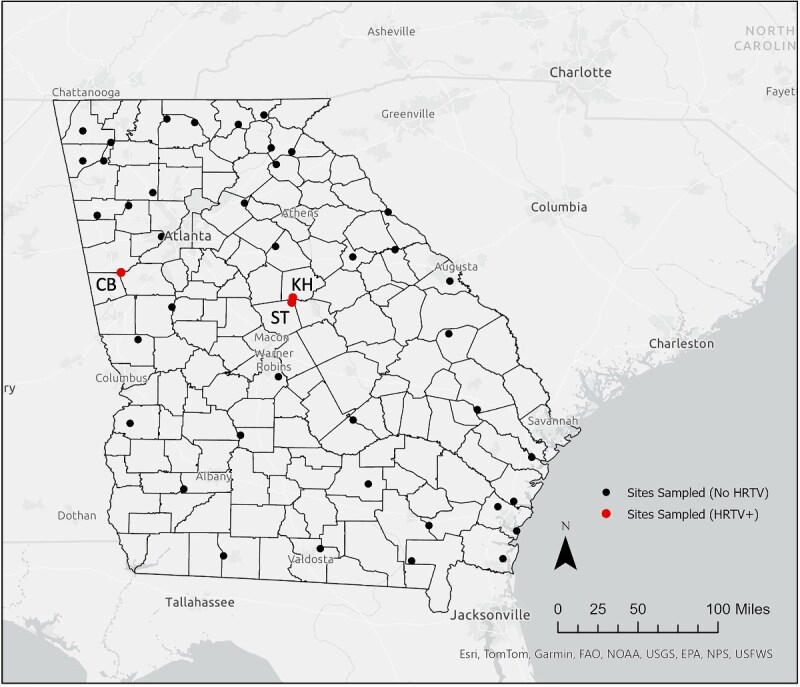
Map of collection sites visited from 2021 to 2023**.** State and county boundaries for Georgia are highlighted in black. Black dots represent locations sampled where HRTV-positive pools of ticks were not found. Red dots represent locations where HRTV-positive pools were found with each location labelled with its two-letter site code (KH, KH road site; ST, ST road site; CB, Chattahoochee Bend State Park). Though no positive pools of HRTV were found from 2021 to 2023, KH is still highlighted due to presence of positive ticks from 2019 detected by our group as originally documented in Romer *et al*. 2022 and described in Table 2. Map created in ArcGIS pro v3.1.0.

In total, 21 386 ticks were collected across the 3-year period: 7687 in 2021, 3444 in 2022, and 10 255 in 2023 (Table 1). Most (20 959, 98%) were *A. americanum* adults or nymphs. Six pools of *A. americanum* ticks tested positive for HRTV: one pool of 19 nymphs from 2021, two pools of 27 nymphs and 28 nymphs from 2022, and 3 pools of 25 nymphs, 5 adult females, and 25 nymphs in 2023 (Table 2). Five of the six HRTV-positive pools were collected at ST road site (ST) in central GA, a small fragmented mixed-forest patch of ~100 m × 400 m between two roads separating a farm and protected forest, and the sixth was collected at Chattahoochee Bend State Park (CB), one of GA’s largest state parks measuring almost 3000 acres (Georgia Department of Natural Resources 2024) and characterized by a variety of forested habitats (Fig. 1). The MIRs calculated for each site and year were 0.33 per 1000 nymphs at ST in 2021, 1.97 per 1000 nymphs at ST in 2022, 0.59 per 1000 nymphs at ST in 2023, 2.56 per 1000 adults at ST in 2023, and 0.46 per 1000 nymphs at CB in 2023.

**Table 1:** Species and life stage of ticks collected during field sampling from 2021 to 2023

**Species**	**Stage**	**2021**	**2022**	**2023**
*Amblyomma americanum*	Adult	1984	600	1109
*Amblyomma americanum*	Nymph	5584	2708	8974
*Dermacentor variabilis*	Adult	100	54	19
*Amblyomma maculatum*	Adult	19	13	14
*Ixodes scapularis*	Adult	0	66	64
*Ixodes* species	Nymph	0	0	8
*Haemaphysalis longicornis*	Nymph	0	3	67
**Total**		7687	3444	10 255

**Table 2:** Heartland positive pool characteristics. All pools consist of *Amblyomma americanum*. Location abbreviations: KH, KH road site; ST, ST road site; CB, Chattahoochee Bend State Park. ^*^2019 HRTV-positive pools were originally published in Romer *et al*. 2022

**Year**	**Lab code**	**Location**	**Date collected**	**Number of ticks**	**Life stage**
2019^*^	23	KH	4/28/19	5	Adult (Female)
2019^*^	26	KH	4/28/19	25	Nymph
2019^*^	504	ST	6/14/19	5	Adult (Male)
2021	404	ST	6/15/21	19	Nymph
2022	100	ST	5/12/22	27	Nymph
2022	187	ST	6/10/22	28	Nymph
2023	56F	ST	5/12/23	25	Nymph
2023	432R	ST	6/16/23	5	Adult (Female)
2023	642T	CB	7/19/23	25	Nymph

### Heartland virus sequences from Georgia are highly related across space and time

Full HRTV genome sequences were generated using our novel HRTV-specific multiplex amplicon protocol, with 88%–100% coverage achieved across all three segments from the six positive tick pools (Supplemental Table S3). Including the three HRTV-positive tick pools collected and sequenced by our group in 2019 from sites ST and KH (Romer *et al*. 2022), the nine GA sequences were highly similar with 99.52%–99.99% nucleotide identity across the entire genome and similar identity relationships across the individual segments (Table 3). The largest genetic differences were seen between the sample collected at site CB in 2023 (Sample 642T) and other samples from GA, collected ~175 km away. Sample 642T differed from the other GA sequences by 44–51 single-nucleotide polymorphisms (SNPs), while the sequences from sites KH and ST differed from one another by only 1–27 SNPs (Table 3). Few nonsynonymous SNPs were found in sample 642T: 4 (of 24 total SNPs) in the L segment, 1 (of 13) in the M segment, and none in the S segment. Interestingly, while three of these nonsynonymous SNPs were unique to sample 642T, two SNPs in the L segment (T168A and R183K) were shared with the other 12 reference sequences obtained from across the country. When comparing all 21 HRTV sequences from the USA, the nucleotide identity ranged from 92.76% to 99.99% across the three segments, equating to between 1 and 782 SNP differences (Supplemental Fig. S2). Across the HRTV genome, the GA sequences shared 70 SNPs (35 in L, 20 in M, 12 in S), 6 of which (1 in L, 2 in M, three in S) were nonsynonymous. While the GA sequences were all derived from tick pools, the 12 available reference sequences from GenBank were from both tick pools (5) and human infections (7). There were no SNPs common to HRTV sequences from only ticks or only humans.

**Table 3:** Pairwise genetic distances between HRTV sequences in Georgia. Number of SNPs and percent nucleotide identity are displayed across the concatenated genome of the three segments excluding gaps.

**Location**		**Site KH**		**Site ST**					
**Site KH**		**23_T_GA_KH_** **2019**	**26_T_GA_KH_** **2019**	**100_T_GA_ST_** **2022**	**404_T_GA_ST_** **2021**	**187_T_GA_ST_** **2022**	**504_T_GA_ST_** **2019**	**056F_T_GA_ST_** **2023**	**432R_T_GA_ST_** **2023**
	**26_T_GA_KH_** **2019**	3 (99.97%)							
**Site ST**	**100_T_GA_ST_** **2022**	10 (99.91%)	7 (99.93%)						
	**404_T_GA_ST_** **2021**	8 (99.92%)	7 (99.93%)	2 (99.98%)					
	**187_T_GA_ST_** **2022**	11 (99.9%)	8 (99.92%)	1 (99.99%)	3 (99.97%)				
	**504_T_GA_ST_** **2019**	23 (99.78%)	22 (99.79%)	23 (99.78%)	21 (99.8%)	24 (99.77%)			
	**056F_T_GA_ST_** **2023**	11 (99.9%)	10 (99.91%)	7 (99.93%)	7 (99.93%)	8 (99.92%)	26 (99.75%)		
	**432R_T_GA_ST_** **2023**	12 (99.89%)	11 (99.9%)	8 (99.92%)	10 (99.91%)	9 (99.91%)	27 (99.74%)	7 (99.93%)	
**Site CB**	**642T_T_GA_CB_2023**	47 (99.56%)	44 (99.58%)	46 (99.56%)	46 (99.56%)	47 (99.56%)	51 (99.52%)	47 (99.56%)	46 (99.56%)

### US Heartland virus sequences demonstrate geographic clustering and potential reassortment

**Figure 2:**
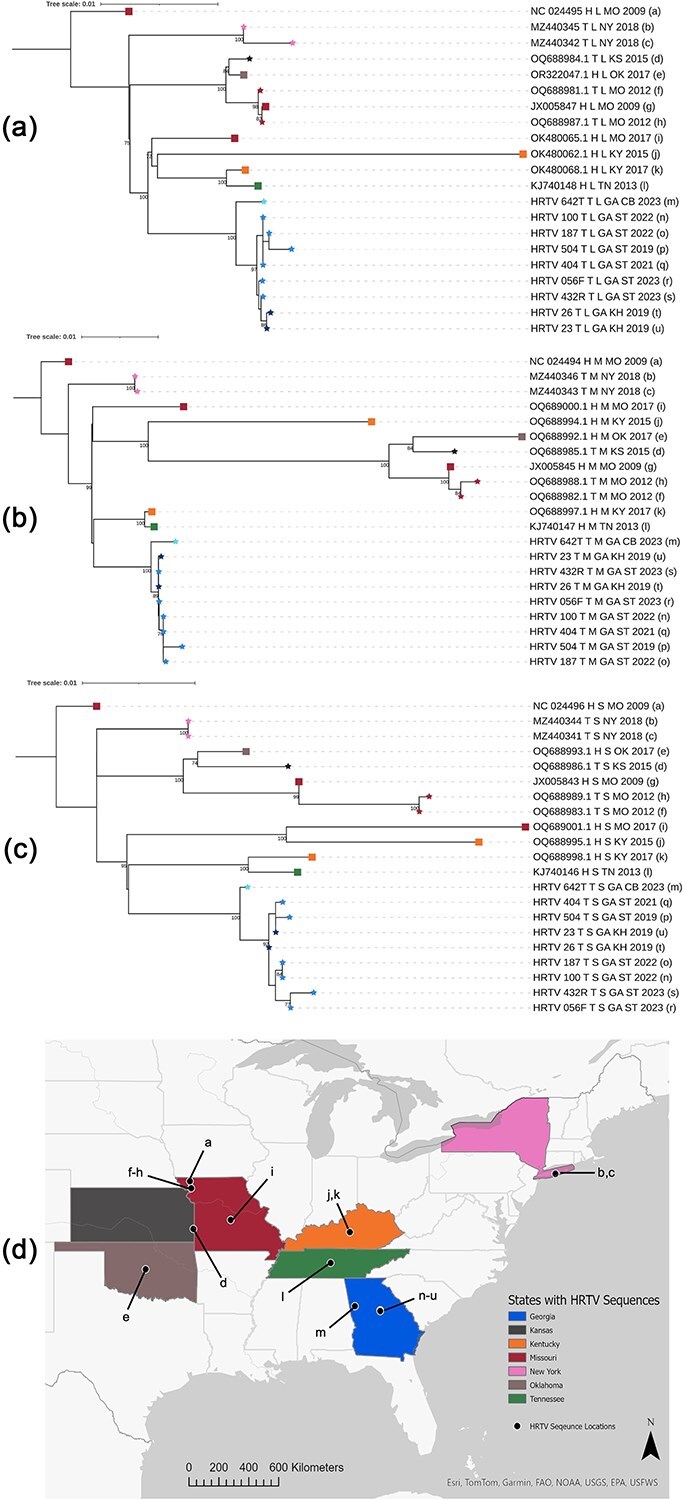
Phylogenetic and geographic relationships of HRTV sequences in the USA**.** Segments were aligned separately, and maximum likelihood trees are displayed for the L segment (a), M segment (b), and S segment (c). Reference sequences from GenBank were named by the accession code followed by T (tick sample) or H (human sample), the segment, the state abbreviation for the collection location, and the year of collection. Sequences generated in this study were named by HRTV, sample number, T, GA, collection site, and year collected. All trees were rooted on the reference sequence from MO patient 1. Ultrafast bootstrap values above 0.9 are displayed on the tree. Tips are labelled with a star for tick samples and a square for human samples. The tip colour indicates the location of collection. (d) A map of the eastern United States with states coloured as in the phylogenetic trees (a–c). Locations for each sequence included are marked with a black dot. Dots are placed where either (1) sequence locations were documented in the literature (Savage *et al*. 2013; Savage *et al*. 2018; Dupuis, 2nd *et al*. 2021) or (2) in the centre of the state/county reported when finer resolution location data was unavailable. Dots are additionally marked with a-u and correspond to the sequences in the tree marked in (a–c). Map created in ArcGIS Pro v3.1.0.

The 21 US HRTV sequences generally clustered geographically on maximum-likelihood phylogenetic trees for each segment (Fig. 2). Across all trees, the GA sequences clustered together, and the sample collected at the western site CB (HRTV 642T) was the most divergent. The two sequences from NY ticks were similarly clustered together across all segments. Furthermore, samples from neighbouring states were generally clustered together (OK/KS/MO and TN/KY) in each segment, regardless of date of sample collection or host. The Mantel test also supported geographic clustering, with a correlation of 0.53 (*P*-value = .001) between genetic distance and spatial distance.

While the overall phylogenetic structure was similar across segments, we observed potential reassortment between segments. Specifically, sequences OQ688994 (M segment), OQ688995 (S segment), and OK480062 (L segment) were collected from a human case in KY in 2015; they clustered with the other KY and TN sequences in the L segment but with the MO, OK, and KS clades in the M segment, and with a single MO sequence in the S segment, all with moderate to high ultrafast bootstrap support (Fig. 2). Visualization of these trees as tanglegrams supported reassortment of the M segment for this sample (Supplemental Fig. S3, sample labelled J). RDP4 was also used to assess for reassortment events between the HRTV segments and did not identify any events with high certainty. Ongoing genomic surveillance of HRTV is needed to better understand the potential for reassortment, as has been reported for the related Dabie bandavirus.

### Contemporary Heartland virus strains in the USA emerged within the last 100 years

**Figure 3:**
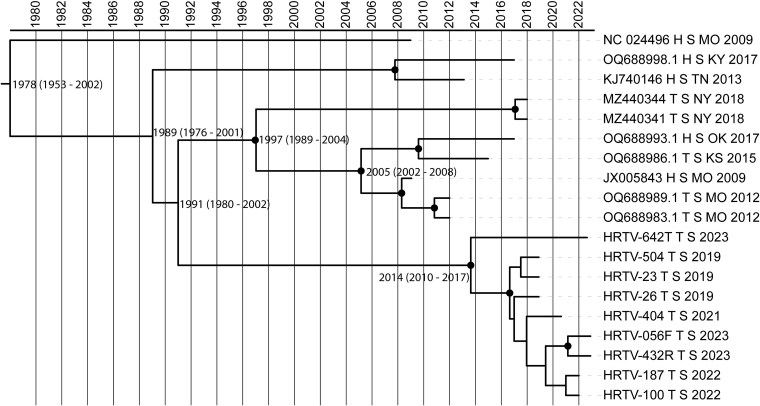
Time-scaled Bayesian phylogenetic analysis of HRTV S segment. Nodes are labelled with median time to recent common ancestor (tMRCA) with [95% HPD]. Black circles indicate nodes with a posterior probability of at least 0.9.

Time-scaled Bayesian phylogenetic analysis showed an evolutionary rate of 5.3 × 10^−4^ substitutions/site/year (s/s/y) in the S segment; this was not performed for the M or L segments given the lack of evidence for temporal signal by BETS. Based on the S segment, the most recent common ancestor (MRCA) for the GA clade was in 2014 (95% HPD 2010–17), and the overall root of the tree was in 1978 (95% HPD 1953–2002) (Fig. 3).

### *A. americanum* ticks feed on a variety of wildlife in Georgia

To begin to evaluate potential reservoir species for HRTV, a total of 12 *A. americanum* pools from GA underwent host bloodmeal analysis using retrotransposon qPCR. Each HRTV-positive pool was matched with an HRTV-negative pool with ticks of the same life stage, collection site, and collection date (Table 4, Supplemental Table S6). The HRTV-positive pools of nymphs from ST (samples 404, 187, 100, and 56F) tested positive for birds, deer, rabbits, and squirrels/chipmunks. Their matched controls from HRTV-negative pools had similar results, with two exceptions: HRTV-negative pool 403 was positive for rabbit, while its matched HRTV-positive pool 404 was not, and HRTV-positive pool 56F was positive for squirrel/chipmunk, while its matched HRTV-negative pool 55E was not. Both pools of adult ticks from site ST tested negative for most hosts, other than rabbit in the HRTV-negative pool. Finally, the nymph pools from CB both tested positive for deer, and the HRTV-negative pool additionally tested positive for squirrels/chipmunks.

**Table 4:** Host bloodmeal PCR results. The following species with no positive results were not included in the table: mice, voles, shrews, skunks, and opossums

**Sample name**	**HRTV status**	**Tick stage (*n*)**	**Location, year**	**Rabbit PCR**	**Bird PCR**	**Squirrel/Chipmunk PCR**	**Deer PCR**
403	**−**	Nymph (25)	ST, 2021	+	+	−	+
404	**+**	Nymph (19)	ST, 2021	−	+	−	+
186	**−**	Nymph (27)	ST, 2022	−	−	−	+
187	**+**	Nymph (28)	ST, 2022	−	−	−	+
99	**−**	Nymph (26)	ST, 2022	+	+	+	+
100	**+**	Nymph (27)	ST, 2022	+	+	+	+
55E	**−**	Nymph (25)	ST, 2023	+	+	−	+
56F	**+**	Nymph (25)	ST, 2023	+	+	+	+
432R	**+**	Adult (5)	ST, 2023	−	−	−	−
433S	**−**	Adult (5)	ST, 2023	+	−	−	−
641S	**−**	Nymph (25)	CB, 2023	−	−	+	+
642 T	**+**	Nymph (25)	CB, 2023	−	−	−	+

## Discussion

We identified a persistent focus of genetically conserved HRTV in central Georgia. We collected over 21 000 ticks across 3 years and found MIRs ranging from 0.33 per 1000 nymphs in 2021 to 2.56 per 1000 adults in 2023, compatible with our prior work (Romer *et al*. 2022) and other studies (Savage *et al*. 2013; Newman *et al.* 2020). Using a novel multiplex amplicon sequencing assay, we generated six new HRTV genome sequences and examined phylogenetic relationships across multiple years and locations.

One key finding was the very high genetic conservation of HRTV from ticks collected at a focal site in central GA across 5 years. Studies of Powassan virus (POWV), an unrelated tick-borne virus, also endemic to the USA, have similarly reported foci of genetically conserved viruses that were stable across multiple years, but have also reported geographic dispersal of closely related POWV strains (Brackney *et al.* 2008; Anderson and Armstrong 2012; McMinn *et al.* 2023; Vogels *et al*. 2023). We did not observe geographic dispersal of closely-related HRTV strains; instead, we observed phylogenetic clustering by location throughout the USA, though it is notable that sequence data are limited, with only 12 reference sequences available from elsewhere in the USA and our 9 from Georgia.

A second key finding was that time-scaled phylogenetic analysis indicated the recent spread of HRTV in the USA, with all 21 sequences sharing a common ancestor within the last 75 years (since the 1950s), and sequences from Georgia sharing a common ancestor within the last 15 years (since 2010). This timeline is compatible with historical patterns of *A. americanum* prevalence. In the 18th century, dense forests supported high populations of deer and associated ticks; however, deforestation and decimation of deer populations in the 19th century led to large-scale *A. americanum* range retraction (Rochlin *et al.* 2022). The resulting fracturing of tick populations could have generated geographically isolated HRTV lineages that diverged over the last hundred years. Supporting this, there is evidence for geographic population structure within *A. americanum* ticks between different regions of the USA (Monzón *et al.* 2016).

Overall, our phylodynamic results for HRTV mirror what has been reported for the closely related Dabie bandavirus (DBV) in Asia. Our estimated evolutionary rate for HRTV, 5.3 × 10^−4^ s/s/y, is similar to the DBV literature, where studies report rates ranging from 1.1 × 10^−4^ to 1.1 × 10^−3^ s/s/y across the three segments, with most estimates around 2 × 10^−4^, and rates generally higher in the S segment compared to M (Fu *et al.* 2016; Liu *et al.* 2016a; Liu *et al.* 2016b; Yun *et al.* 2020a; Liu *et al.* 2023). Similar to our results, many DBV studies have shown distinct genotypes clustering by geographic region (Yoshikawa *et al.* 2015; Liang *et al.* 2023; Liu *et al*. 2023; Zu *et al.* 2024). Interestingly, however, several recent studies also report mixing of DBV genotypes across different countries in Asia (Fu *et al*. 2016; Li *et al*. 2021), which is thought to be due to migratory bird movement carrying the DBV vector, *Haemaphysalis longicornis*, across longer distances (Shi *et al.* 2017).

Despite the small number of HRTV sequences, our phylogenetic analysis suggests the possibility of reassortment between genome segments, a common mechanism of increasing genetic diversity in bunyaviruses and other segmented viruses (Vijaykrishna *et al.* 2015). Reassortment has been frequently described for DBV, allowing new genotypes to emerge after co-infection events (Lv *et al.* 2017; Yun *et al.* 2020b; Li *et al*. 2021; Wu *et al.* 2021). This is important because reassorted viruses have the ability to overcome host barriers and have the potential to generate strains of higher pathogenicity as seen in the influenza virus (Smith *et al.* 2009; Vijaykrishna *et al*. 2015). However, because reassortment requires multiple viruses to infect the same cell, it would be expected to occur less frequently in viruses with low prevalence, as HRTV seems to be, based on our study and others. Thus, further surveillance and genome sequencing are needed to evaluate HRTV prevalence and genetic diversity throughout the USA, to more rigorously assess the potential role of reassortment.

Our observation of high HRTV genetic conservation at a focal site across multiple years suggests several possible hypotheses about the enzootic cycle of HRTV, which remains largely unknown (Brault *et al*. 2018). One possibility is that closely related viruses may be sustained within the population through transovarial transmission (also referred to as vertical transmission), as has been documented in experimental studies of HRTV (Godsey *et al.* 2016). In this scenario, infected female *A. americanum* would lay eggs at the site, passing on the virus to their larvae, which would feed on small-range hosts and remain within the site to moult and be captured as nymphs the following year. Vertical transmission typically augments other transmission mechanisms and has not been proposed as a sole mechanism for persistence of other tick-borne viruses (Maqbool *et al.* 2022). Testing larval populations for HRTV is an important next step in investigating this mechanism.

A second potential mechanism for HRTV focality involves propagation of virus through cofeeding transmission, which has also been documented experimentally for HRTV (Godsey *et al*. 2016) and is seen in other tick-borne viruses such as tick-borne encephalitis virus (TBEV) (Labuda *et al.* 1996) and POWV (Obellianne *et al.* 2024). In this case, infected *A. americanum* feed next to uninfected ticks, leading to HRTV transmission through infection of local skin and leukocytes. Co-feeding transmission provides a role for hosts that are not competent for viremic infection themselves, as demonstrated for *Ixodes* species infected with *Borrelia* that feed on hosts whose innate complement systems prevent systemic infection (Voordouw 2015). Co-feeding on hosts with a limited range could explain the persistence of highly related viruses across multiple years.

Finally, HRTV could propagate through horizontal transmission by ticks feeding on infected reservoir hosts. Serosurveys and experimental infections investigating potential HRTV reservoir hosts have not yet identified any candidates (Bosco-Lauth *et al.* 2015; Bosco-Lauth *et al.* 2016). Evidence of seropositivity has been found in multiple wildlife species with white-tailed deer, raccoons, horses, and dogs showing the highest rates of neutralizing antibodies across studies (Riemersma and Komar 2015; Brault *et al*. 2018; Clarke *et al*. 2018b). However, experimental infection of multiple wildlife hosts including white-tailed deer, raccoons, goats, chickens, rabbits, hamsters, and C57BL/6 mice has failed to produce detectable viremia, except in an interferon receptor–deficient mouse model (Bosco-Lauth *et al*. 2016; Clarke *et al.* 2018a). Together, this information suggests that these animals may be incidental hosts, and there is no evidence yet for a viremic reservoir host to support horizontal transmission of HRTV. If there is a role for horizontal or cofeeding transmission in the HRTV enzootic cycle, our HRTV phylogenetic analysis suggests the importance of geographically restricted hosts.

Our preliminary host bloodmeal analysis suggests avenues for further investigation into HRTV enzootic transmission. For example, we detected HRTV in two pools of nymphs that had only fed on deer, which do not support HRTV viremia in experimental infection studies (Clarke *et al*. 2018a). This suggests that the nymphs had acquired HRTV through vertical transmission, co-feeding transmission, or viremic transmission from a reservoir host that was not tested in our initial bloodmeal analysis. We also detected HRTV in pools of ticks that had fed on rabbits (which do not support HRTV viremia in experimental infection studies) (Bosco-Lauth *et al*. 2016), as well as squirrels/chipmunks and birds (which have not been evaluated in experimental infection studies). While we did not observe a clear difference in bloodmeal results between HRTV-positive and negative samples, this analysis was limited by our analysis of tick pools rather than individual ticks, as it is unclear whether the host bloodmeal was present in HRTV-positive tick(s) or HRTV-negative tick(s) within the pool; for the same reason, interpretation of *C*_T_ values in pooled samples is challenging. Finally, although nine potential hosts were tested in this initial study, it is possible that other, yet-untested hosts are important to HRTV transmission ecology, an important future direction for this work.

In conclusion, through extensive field work and the use of a novel multiplex amplicon sequencing assay, we have gained important insight into HRTV diversity and evolution. Key findings are that HRTV sequences from a focal site in GA were highly related across time; that there is strong geographic clustering of HRTV across the USA with this attribute being more important than host or year; and that all available HRTV sequences share a common ancestor within the recent past, suggesting that contemporary strains of HRTV emerged within the last 100 years. Our work provides a foundation for further investigation into the HRTV enzootic cycle and spatial structure across the USA.

## Supplementary Material

HRTVFocalPersistanceSupplementFiles_veaf064

HRTVFocalPersistenceSupplemental_Tables_veaf064

## Data Availability

HRTV genome sequences are available on NCBI with GenBank accession numbers listed in Table S3.
